# Molecular detection of foodborne pathogens in Ardabil’s milk supply

**DOI:** 10.1186/s12917-025-05018-x

**Published:** 2025-10-02

**Authors:** Faraz Malek Bagali, Eslam Moradi-Asl, Abbas Abbasi-Ghahramanloo, Amirhossein Sahebkar, Farzad Khademi

**Affiliations:** 1 Department of Microbiology, School of Medicine, Ardabil University of Medical Sciences, Ardabil, Iran; 2 Department of Public Health, School of Health, Ardabil University of Medical Sciences, Ardabil, Iran; 3 Arthropod-Borne Diseases Research Center, Ardabil University of Medical Sciences, Ardabil, Iran; 4 Center for Global Health Research, Saveetha Institute of Medical and Technical Sciences, Saveetha Medical College and Hospitals, Saveetha University, Chennai, India; 5 Biotechnology Research Center, Pharmaceutical Technology Institute, Mashhad University of Medical Sciences, Mashhad, Iran; 6 Applied Biomedical Research Center, Basic Sciences Research Institute, Mashhad University of Medical Sciences, Mashhad, Iran

**Keywords:** Foodborne diseases, Milk, Frequency, Public health, Polymerase chain reaction

## Abstract

**Background:**

Foodborne diseases, often associated with animal products, cause illnesses globally. Contaminated animal products, particularly milk, are responsible for two-thirds of foodborne disease outbreaks, posing significant challenges to public health and economic sectors. This highlights the need for stringent food safety measures to protect public health. This descriptive cross-sectional study aimed to investigate the frequency of major foodborne pathogens in bulk milk samples in Ardabil province, Iran.

**Methods:**

Between April and August 2024, 281 unpasteurized bulk milk samples were collected from cattle, sheep, and goat in Ardabil province, Iran. Samples were transported under hygienic conditions and stored at -20°C. DNA was extracted from 15 mL of milk samples and then molecular identification of various foodborne pathogens was performed using polymerase chain reaction (PCR) and nested PCR methods. Geographic information systems (GIS) were employed to determine pathogen dispersion, creating scatter plots using ArcMap 10.8.1 software based on GPS coordinates of sampling locations.

**Results:**

Among 281 bulk milk specimens that were tested, the frequency of various foodborne pathogens was as follows: *Coxiella burnetii* 9.2%, *Listeria monocytogenes* 1%, *Brucella* spp. 11.3%, *Campylobacter jejuni* 7.8%, *Mycobacterium tuberculosis* complex 8.1%, *Salmonella enterica* 6.4%, *Staphylococcus aureus* 3.9%, *Escherichia coli* 3.2%, and *Bacillus cereus* 12.8%.

**Conclusions:**

This study highlights the high frequency of major foodborne pathogens in unpasteurized bulk milk samples from Ardabil province, Iran. This underscores the critical need for enhanced food safety measures. The high frequency of contamination, particularly from *B. cereus*, *Brucella* spp., and *C. burnetii*, pose serious public health risks. Implementing stringent monitoring and control strategies in the dairy industry is essential to reduce the incidence of foodborne diseases and protect consumers. Continuous surveillance and education on proper food handling practices are imperative to safeguard public health and prevent future outbreaks of foodborne illnesses in the region.

## Background

Foodborne diseases, commonly referred to as food poisoning, are associated with consuming foods, particularly animal products, and can lead to public health challenges. Animal-derived foods can become contaminated with harmful microorganisms like bacteria, viruses, parasites, fungi or by various chemicals during processing, slaughter, storage, and packaging. Bacteria, in particular, are responsible for approximately two-thirds of foodborne disease outbreaks [1–3]. Key bacterial pathogens, including *Coxiella burnetii* (*C. burnetii*), *Listeria monocytogenes* (*L. monocytogenes*), *Campylobacter jejuni* (*C. jejuni*), *Staphylococcus aureus* (*S. aureus*), *Brucella* spp., *Salmonella* spp., and *Mycobacterium tuberculosis* (*M. tuberculosis*) complex, represent significant foodborne threats and pose major public health challenges globally [4]. These pathogens frequently cause diarrhea, the most common foodborne illness, but they can also result in severe conditions like kidney and liver failure, neurological disorders, reactive arthritis, and even death [5]. The World Health Organization (WHO) has reported that foodborne and waterborne diarrheal diseases kill 2.2 million people annually, with children making up the majority of these fatalities [5]. In low- and middle-income countries, unsafe food consumption leads to an annual economic loss of $110 billion due to reduced productivity and medical expenses. Children under five years of age are especially vulnerable, bearing a significant portion of the disease burden, resulting in 125,000 deaths each year https://www.who.int/news-room/fact-sheets/detail/food-safety. Based on the water- and foodborne disease outbreak reports from the Center of Disease Control of the Ministry of Health in Iran, a total of 4,490 outbreaks of water- and foodborne diseases along with 31 deaths were reported in the country in 2023, which show an approximately 13.5% growth compared with 2022 (https://icdc.behdasht.gov.ir/Outbreak_water_food_status/). This issue underscores the importance of implementing stringent food safety measures and practices to prevent contamination and protect public health.

Milk, a widely consumed animal-derived food, serves as a frequent source of infection across different social groups. Contaminated dairy products contribute to approximately 4% of global foodborne illnesses, affecting people of all ages and occupations. During milking, improper handling or exposure to infected animals can introduce harmful bacteria into raw milk. Unlike pasteurized milk, which undergoes heat treatment to eliminate pathogens, raw milk retains these microorganisms, increasing health risks. Fortunately, implementing basic safety measures can significantly reduce the likelihood of milkborne diseases [6].

**Fig. 1:**
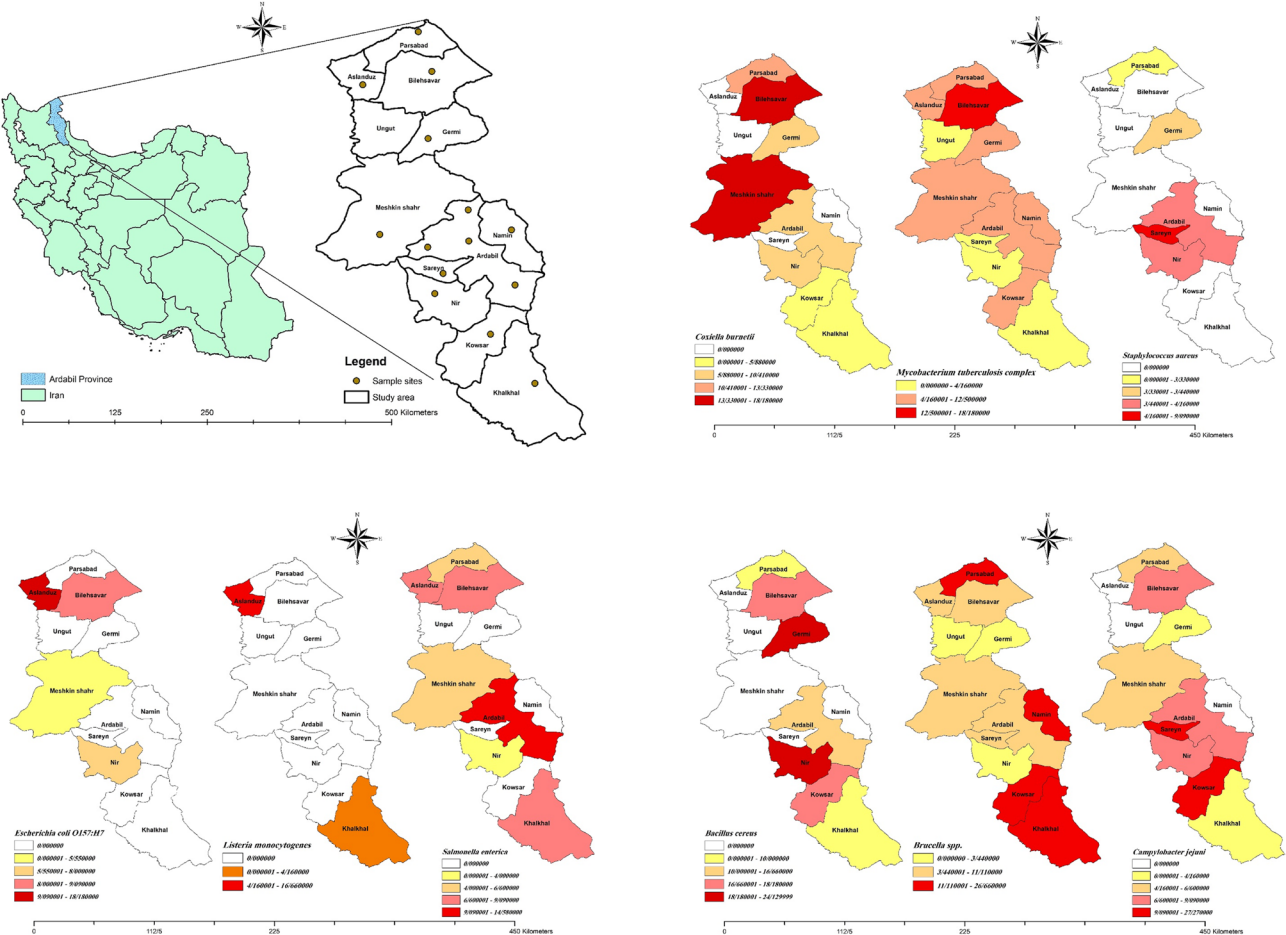
Geographical distribution of foodborne pathogens in bulk milk samples in Ardabil province

Ardabil, located in northwest Iran (Fig. 1), is an important region for agriculture and livestock farming, producing a variety of crops and dairy products, including milk. According to research conducted in Ardabil city, 51.67% of residents consumed pasteurized milk, 40.67% opted for fresh bulk milk, and 4.67% used both types. Meanwhile, 3% reported no milk consumption. Given these consumption patterns, enhancing public awareness regarding the prevalence of milkborne pathogens, particularly in bulk milk, is crucial for safeguarding consumer health [7]. Following this, proper milk handling, storage, and preparation practices should be implemented to minimize the risk of milkborne illnesses. Given the absence of studies on the frequency of milkborne bacteria in Ardabil province, this descriptive cross-sectional study was conducted to investigate the frequency of major milkborne pathogens in bulk milk samples from the region.

## Methods

### Milk collection

In this cross-sectional study carried out from April to August 2024, a total of 281 unpasteurized bulk milk samples were obtained from traditional livestock farms across various cities in Ardabil province, Iran. In this study, the sample size was estimated based on previous reports indicating that approximately 10% of milk samples tested positive (*p* = 0.10). Considering a type, I error (α) of 5% and a desired precision of 3.5%, the required sample size was calculated to be 281 samples. Proportion sample size formula:

$$\:n=\frac{{z}_{1-\frac{\alpha\:}{2}}^{2}\times\:p\left(1-p\right)}{{d}^{2}}$$

where Z_{1-α/2} = 1.96 for a 95% confidence level, α = 0.05, and d = 0.035 (desired precision), the calculated sample size was 281 samples. This approach ensured adequate statistical power to detect the expected prevalence with the specified margin of error. To conduct the sampling, eligible farms was selected based on predefined criteria: each farm had to have at least one lactating animal (cattle, sheep, or goat), adhere to traditional livestock practices, and consent to participate in the study. Farms were selected using a stratified random sampling approach to ensure representation across different geographic regions. Within each selected farm, healthy lactating animals were chosen through simple random sampling. Animals were included if they were clinically healthy, actively lactating, and had not received any antibiotic. Farms that did not meet these conditions were excluded from the study. From each selected farm, a total of 50 mL milk sample was collected under hygienic protocols. In total, samples were obtained from 138 cattle, 113 sheep, and 30 goat farms. These samples were assessed for physical properties such as color, pH, and density. Subsequently, they were transported in cool boxes with ice packs to the microbiology research laboratory and stored at − 20 °C until genomic analysis.

## Total DNA isolation

The frozen raw bulk milk samples were thawed at room temperature and utilized for total DNA extraction following a modified version of the Jeršek et al.. protocol [8]. Briefly, 15 mL of each sample was initially heated for 20 min, then incubated at −20 °C for 5 min, and centrifuged at 6,000*g* for 5 min to separate the skim milk from the cream. The resulting precipitates were mixed with 2 mL of buffer (containing 6% SDS, 5% DMSO, acetone, 5 g MgCl_2_, and 10 g sodium acetate per liter, adjusted to pH 7.2), and incubated at 72 °C for 1 h. Afterwards, the mixture was centrifuged at 6,000*g* for 10 min. The supernatants were discarded, and the precipitates were washed with 1 mL of phosphate-buffered saline (PBS) solution. The precipitates were then used for DNA extraction using a total DNA isolation solution (Qiagen, Germany), and stored at −20 °C until needed for polymerase chain reaction (PCR) amplification.

## PCR amplification

Molecular identification of different foodborne pathogens, i.e.,* C. burnetii*, *L. monocytogens*, *Brucella* spp., *C. jejuni*, *M. tuberculosis* complex, *S. enterica*, *S. aureus*, *E. coli*, and *B. cereus*, in raw bulk milk samples was performed using the PCR and Nested PCR methods (Eppendorf thermal cycler, Germany). The specific gene primer sequences (Metabion, Germany) used in this study for microorganism detection, along with the PCR conditions, are detailed in Table 1. Amplifications were performed in a total volume of 15 µL, comprising 12 µL of Master Mix, 2 µL of template DNA (50 ng/µL), and 1 µL of each primer (10 µmol/L) (Ampliqon, Denmark). DNA amplification products were separated on a 1% agarose gel (Electrophoresis, Padideh Nojen Pars, Iran) and visualized under ultraviolet light. A distinct, sharply defined single band—corresponding to the expected amplicon size and free of non-specific products—was observed for a selected isolate. This sample was submitted to Pishgam Biotech (Iran) for sequencing via the Sanger method. The acquired sequence was analyzed using Chromas Lite (version 2.6.6), and homology was assessed through BLAST searches against entries in the NCBI GenBank database to confirm sequence identity and target accuracy.

**Table 1:** Used primers along with PCR programs

Bacterial pathogen	Target gene	Oligonucleotide sequence (5′ to 3′)	PCR condition	Amplicon size (bp)	Reference
*C. burnetii*	*com1*	F: AGTAGAAGCATCCCAAGCATTG R: TGCCTGCTAGCTGTAACGATTG	Initial denaturation at 95 °C for 5 min (1 cycle) $$\left.\begin{array}{r}\mathrm{Denaturation}\;\mathrm{at}\;94\;^\circ\mathrm C\;\mathrm{for}\;1\;\min\\\mathrm{Annealing}\;\mathrm{at}\;60\;^\circ\mathrm C\;\mathrm{for}\;1\;\min\\\mathrm{Extension}\;\mathrm{at}\;72\;^\circ\mathrm C\;\mathrm{for}\;1\;\min\end{array}\right\}34\;\mathrm{cycles}$$	501	[4]
	*com2*	F: GAAGCGCAACAAGAAGAACAC R: TTGGAAGTTATCACGCAGTTG	Initial denaturation at 95 °C for 5 min (1 cycle) $$\left.\begin{array}{r}\mathrm{Denaturation}\;\mathrm{at}\;94\;^\circ\mathrm C\;\mathrm{for}\;1\;\min\\\mathrm{Annealing}\;\mathrm{at}\;57\;^\circ\mathrm C\;\mathrm{for}\;1\;\min\\\mathrm{Extension}\;\mathrm{at}\;72\;^\circ\mathrm C\;\mathrm{for}\;1\;\min\end{array}\right\}34\;\mathrm{cycles}$$	438	
*L. monocytogens*	*hlyA*	F: ATCATCGACGGCAACCTCGGAGAC R: CACCATTCCCAAGCTAAACCAGTGC	Initial denaturation at 95 °C for 5 min (1 cycle) $$\left.\begin{array}{r}\mathrm{Denaturation}\;\mathrm{at}\;94\;^\circ\mathrm C\;\mathrm{for}\;1\;\min\\\mathrm{Annealing}\;\mathrm{at}\;67\;^\circ\mathrm C\;\mathrm{for}\;45\;\sec\\\mathrm{Extension}\;\mathrm{at}\;72\;^\circ\mathrm C\;\mathrm{for}\;1\;\min\end{array}\right\}34\;\mathrm{cycles}$$	404	[9]
*Brucella* spp.	*bcsp31*	F: TGGCTCGGTTGCCAATATCAA R: CGCGCTTGCCTTTCAGGTCTG	Initial denaturation at 95 °C for 5 min (1 cycle) $$\left.\begin{array}{r}\mathrm{Denaturation}\;\mathrm{at}\;94\;^\circ\mathrm C\;\mathrm{for}\;1\;\min\\\mathrm{Annealing}\;\mathrm{at}\;59\;^\circ\mathrm C\;\mathrm{for}\;45\;\sec\\\mathrm{Extension}\;\mathrm{at}\;72\;^\circ\mathrm C\;\mathrm{for}\;1\;\min\end{array}\right\}34\;\mathrm{cycles}$$	223	[4]
*C. jejuni*	*hipO*	F: GAAGAGGGTTTGGGTGGTG R: AGCTAGCTTCGCATAATAACTTG	Initial denaturation at 95 °C for 5 min (1 cycle) $$\left.\begin{array}{r}\mathrm{Denaturation}\;\mathrm{at}\;94\;^\circ\mathrm C\;\mathrm{for}\;1\;\min\\\mathrm{Annealing}\;\mathrm{at}\;57\;^\circ\mathrm C\;\mathrm{for}\;45\;\sec\\\mathrm{Extension}\;\mathrm{at}\;72\;^\circ\mathrm C\;\mathrm{for}\;1\;\min\end{array}\right\}34\;\mathrm{cycles}$$	735	[10]
*M. tuberculosis complex*	*IS6110*	F: CGTGAGGGCATCGAGGTGGC R: GCGTAGGCGTCGGTGACAAA	Initial denaturation at 95 °C for 5 min (1 cycle) $$\left.\begin{array}{r}\mathrm{Denaturation}\;\mathrm{at}\;94\;^\circ\mathrm C\;\mathrm{for}\;1\;\min\\\mathrm{Annealing}\;\mathrm{at}\;64\;^\circ\mathrm C\;\mathrm{for}\;45\;\sec\\\mathrm{Extension}\;\mathrm{at}\;72\;^\circ\mathrm C\;\mathrm{for}\;1\;\min\end{array}\right\}34\;\mathrm{cycles}$$	245	[11]
*S. enterica*	*invA*	F: AATTATCGCCACGTTCGGGCAA R: TCGCACCGTCAAAGGAACC	Initial denaturation at 95 °C for 5 min (1 cycle) $$\left.\begin{array}{r}\mathrm{Denaturation}\;\mathrm{at}\;94\;^\circ\mathrm C\;\mathrm{for}\;1\;\min\\\mathrm{Annealing}\;\mathrm{at}\;64\;^\circ\mathrm C\;\mathrm{for}\;30\;\sec\\\mathrm{Extension}\;\mathrm{at}\;72\;^\circ\mathrm C\;\mathrm{for}\;1\;\min\end{array}\right\}34\;\mathrm{cycles}$$	278	[12]
*S. aureus*	*sea*	F: TGTATGTATGGAGGTGTAAC R: ATTAACCGAAGGTTCTGT	Initial denaturation at 95 °C for 5 min (1 cycle) $$\left.\begin{array}{r}\mathrm{Denaturation}\;\mathrm{at}\;94\;^\circ\mathrm C\;\mathrm{for}\;1\;\min\\\mathrm{Annealing}\;\mathrm{at}\;64\;^\circ\mathrm C\;\mathrm{for}\;30\;\sec\\\mathrm{Extension}\;\mathrm{at}\;72\;^\circ\mathrm C\;\mathrm{for}\;1\;\min\end{array}\right\}34\;\mathrm{cycles}$$	270	[13]
*E. coli*	*eaeA*	F: TGAGCGCCCAGCAAATGGCT R: TGTGCGCTTTGGCTTCCGCT	Initial denaturation at 95 °C for 5 min (1 cycle) $$\left.\begin{array}{r}\mathrm{Denaturation}\;\mathrm{at}\;94\;^\circ\mathrm C\;\mathrm{for}\;1\;\min\\\mathrm{Annealing}\;\mathrm{at}\;64\;^\circ\mathrm C\;\mathrm{for}\;30\;\sec\\\mathrm{Extension}\;\mathrm{at}\;72\;^\circ\mathrm C\;\mathrm{for}\;1\;\min\end{array}\right\}34\;\mathrm{cycles}$$	555	[14]
*B. cereus*	*Bal*	F: TGCAACTGTATTAGCACAAGCT R: TACCACGAAGTTTGTTCACTACT	Initial denaturation at 95 °C for 5 min (1 cycle) $$\left.\begin{array}{r}\mathrm{Denaturation}\;\mathrm{at}\;94\;^\circ\mathrm C\;\mathrm{for}\;1\;\min\\\mathrm{Annealing}\;\mathrm{at}\;58\;^\circ\mathrm C\;\mathrm{for}\;45\;\sec\\\mathrm{Extension}\;\mathrm{at}\;72\;^\circ\mathrm C\;\mathrm{for}\;1\;\min\end{array}\right\}34\;\mathrm{cycles}$$	533	[15]

## Geographic information systems (GIS)

To determine the percentage of dispersion, scatter plots were created using ArcMap 10.8.1 software. First, the coordinates of the sampling location were measured using GPS, including latitude and longitude. These points were then entered into Excel and imported as point features into ArcMap, specifying the points on the shape file layer of Ardabil province.

### Statistical analysis

Statistical analyses were conducted using Chi-square and Fisher’s exact tests. All computations were performed in SPSS version 16, with a significance level of *p* < 0.05.

## Results

Among 281 unpasteurized bulk milk specimens that were tested, the frequency of various foodborne pathogens was as follows: *C. burnetii* 9.2%, *L. monocytogenes* 1%, *Brucella* spp. 11.3%, *C. jejuni* 7.8%, *M. tuberculosis* complex 8.1%, *S. enterica* 6.4%, *S. aureus* 3.9%, *E. coli* 3.2%, and *B. cereus* 12.8%. The GenBank accession numbers for our nucleotide sequences range from PV344576 to PV344581. The frequency of foodborne pathogens based on specimen type was presented in Table 2.

**Table 2:** The frequency of foodborne pathogens in Raw bulk milk samples in ardabil Province

Microorganisms	Specimens				
	Cattle % (*n*)	Sheep % (*n*)	Goat % (*n*)	*p*-value	Total % (*n*)
*C. burnetii*	9.4% (13 of 138)	7.9% (9 of 113)	13.3% (4 of 30)	0.663	9.2% (26 of 281)
*L. monocytogens*	1.4% (2 of 138)	0.8% (1 of 113)	0% (0 of 30)	1.000	1% (3 of 281)
*Brucella* spp.	10.8% (15 of 138)	12.3% (14 of 113)	10% (3 of 30)	0.902	11.3% (32 of 281)
*C. jejuni*	5.7% (8 of 138)	10.6% (12 of 113)	6.6% (2 of 30)	0.356	7.8% (22 of 281)
*M. tuberculosis complex*	11.5% (16 of 138)	6.1% (7 of 113)	0% (0 of 30)	0.067	8.1% (23 of 281)
*S. enterica*	10.1% (14 of 138)	3.5% (4 of 113)	0% (0 of 30)	0.033	6.4% (18 of 281)
*S. aureus*	5.7% (8 of 138)	1.7% (2 of 113)	3.3% (1 of 30)	0.279	3.9% (11 of 281)
*E. coli*	4.3% (6 of 138)	2.6% (3 of 113)	0% (0 of 30)	0.620	3.2% (9 of 281)
*B. cereus*	13.7% (19 of 138)	9.7% (11 of 113)	20% (6 of 30)	0.292	12.8% (36 of 281)

The geographical distribution of the frequency of foodborne pathogens was shown in Fig. 1; Table 3. The highest frequency of foodborne pathogens isolated from milk samples in different cities of Ardabil province was as follows: *C. burnetii* in Bilehsavar (9, 34.6%), and Meshkin shahr (6, 23%), *L. monocytogenes* in Aslanduz (2, 66.6%), *Brucella* spp. in Khalkhal (6, 18.7%), Parsabad (6, 18.7%), and Kowsar (5, 15.6%), *C. jejuni* in Kowsar (7, 31.8%), and Sareyn (5, 22.7%), *M. tuberculosis* complex in Bilehsavar (11, 47.8%), *S. enterica* in Ardabil (6, 33.3%), *S. aureus* in Sareyn (4, 36.3%), *E. coli* in Aslanduz (4, 44.4%), and *B. cereus* in Germi (10, 27.7%), and Nir (13, 36.1%).

**Table 3:** Frequency of foodborne pathogens detected in Raw bulk milk samples across various areas of ardabil Province

Region	Pathogen type								
	C. burnetii % (*n*)	L. monocytogens % (*n*)	Brucella spp. % (*n*)	C. jejuni % (*n*)	M. tuberculosis complex % (*n*)	S. enterica % (*n*)	S. aureus % (*n*)	E. coli % (*n*)	B. cereus % (*n*)
Ardabil	Cattle: 6.6% (1 of 15) Sheep: 8.3% (1 of 12) Goat: 0	Cattle: 0 Sheep: 0 Goat: 0	Cattle: 6.6% (1 of 15) Sheep: 8.3% (1 of 12) Goat: 0	Cattle: 6.6% (1 of 15) Sheep: 16.6% (2 of 12) Goat: 0	Cattle: 13.3% (2 of 15) Sheep: 0 Goat: 0	Cattle: 26.6% (4 of 15) Sheep: 16.6% (2 of 12) Goat: 0	Cattle: 6.6% (1 of 15) Sheep: 0 Goat: 33.3% (1 of 3)	Cattle: 0 Sheep: 0 Goat: 0	Cattle: 6.6% (1 of 15) Sheep: 8.3% (1 of 12) Goat: 0
Aslanduz	Cattle: 0 Sheep: 0 Goat: 0	Cattle: 14.2% (2 of 14) Sheep: 0 Goat: 0	Cattle: 7.1% (1 of 14) Sheep: 20% (1 of 5) Goat: 0	Cattle: 0 Sheep: 0 Goat: 0	Cattle: 14.2% (2 of 14) Sheep: 0 Goat: 0	Cattle: 14.2% (2 of 14) Sheep: 0 Goat: 0	Cattle: 0 Sheep: 0 Goat: 0	Cattle: 21.4% (3 of 14) Sheep: 20% (1 of 5) Goat: 0	Cattle: 0 Sheep: 0 Goat: 0
Bilehsavar	Cattle: 12% (3 of 25) Sheep: 15% (3 of 20) Goat: 75% (3 of 4)	Cattle: 0 Sheep: 0 Goat: 0	Cattle: 4% (1 of 25) Sheep: 5% (1 of 20) Goat: 0	Cattle: 0 Sheep: 5% (1 of 20) Goat: 0	Cattle: 24% (6 of 25) Sheep: 25% (5 of 20) Goat: 0	Cattle: 8% (2 of 25) Sheep: 5% (1 of 20) Goat: 0	Cattle: 0 Sheep: 0 Goat: 0	Cattle: 4% (2 of 25) Sheep: 5% (1 of 20) Goat: 0	Cattle: 4% (1 of 25) Sheep: 5% (1 of 20) Goat: 75% (1 of 4)
Germi	Cattle: 5% (1 of 20) Sheep: 9% (1 of 11) Goat: 0	Cattle: 0 Sheep: 0 Goat: 0	Cattle: 0 Sheep: 9% (1 of 11) Goat: 0	Cattle: 0 Sheep: 9% (1 of 11) Goat: 0	Cattle: 5% (1 of 20) Sheep: 0 Goat: 0	Cattle: 0 Sheep: 0 Goat: 0	Cattle: 5% (1 of 20) Sheep: 9% (1 of 11) Goat: 0	Cattle: 0 Sheep: 0 Goat: 0	Cattle: 30% (6 of 20) Sheep: 27.2% (3 of 11) Goat: 50% (1of 2)
Khalkhal	Cattle: 10% (1 of 10) Sheep: 11.1% (1 of 9) Goat: 0	Cattle: 0 Sheep: 11.1% (1 of 9) Goat: 0	Cattle: 40% (4 of 10) Sheep: 11.1% (1 of 9) Goat: 100% (1 of 1)	Cattle:0 Sheep: 11.1% (1 of 9) Goat:0	Cattle: 0 Sheep: 11.1% (1 of 9) Goat: 0	Cattle: 10% (1 of 10) Sheep: 11.1% (1 of 9) Goat: 0	Cattle: 0 Sheep: 0 Goat: 0	Cattle: 0 Sheep: 11.1% (1 of 9) Goat: 0	Cattle: 20% (2 of 10) Sheep: 0 Goat: 0
Kowsar	Cattle: 7.6% (1 of 13) Sheep: 10% (1 of 10) Goat:0	Cattle: 0 Sheep: 0 Goat: 0	Cattle: 23% (3 of 13) Sheep: 20% (2 of 10) Goat: 0	Cattle: 30.7% (4 of 13) Sheep: 20% (2 of 10) Goat: 33.3% (1of 3)	Cattle: 7.6% (1 of 13) Sheep: 0 Goat: 0	Cattle: 0 Sheep: 0 Goat: 0	Cattle: 0 Sheep: 0 Goat: 0	Cattle: 0 Sheep: 0 Goat: 0	Cattle: 20% (2 of 10) Sheep: 20% (2 of 10) Goat: 33.3% (1 of 3)
Meshkin shahr	Cattle: 44.4% (4 of 9) Sheep: 25% (2 of 8) Goat: 0	Cattle: 0 Sheep: 0 Goat: 0	Cattle: 0 Sheep: 12.5% (1 of 8) Goat: 0	Cattle: 0 Sheep: 12.5% (1 of 8) Goat: 0	Cattle: 11.1% (1 of 9) Sheep: 0 Goat: 0	Cattle: 22.2% (2 of 9) Sheep: 0 Goat: 0	Cattle: 0 Sheep: 0 Goat: 0	Cattle: 0 Sheep: 0 Goat: 0	Cattle: 0 Sheep: 0 Goat: 0
Namin	Cattle: 0 Sheep: 0 Goat: 0	Cattle: 0 Sheep: 0 Goat: 0	Cattle: 20% (1 of 5) Sheep: 28.5% (2 of 7) Goat: 0	Cattle: 0 Sheep: 0 Goat: 0	Cattle: 20% (1 of 5) Sheep: 0 Goat: 0	Cattle: 0 Sheep: 0 Goat: 0	Cattle:0 Sheep: 0 Goat: 0	Cattle: 0 Sheep: 0 Goat: 0	Cattle: 0 Sheep: 0 Goat: 0
Nir	Cattle: 10% (1 of 10) Sheep: 0 Goat: 0	Cattle: 0 Sheep: 0 Goat: 0	Cattle: 0 Sheep: 0 Goat: 20% (1 of 5)	Cattle: 0 Sheep: 20% (2 of 10) Goat: 0	Cattle: 0 Sheep: 10% (1 of 10) Goat: 0	Cattle: 10% (1 of 10) Sheep: 0 Goat: 0	Cattle: 20% (2 of 10) Sheep: 0 Goat: 0	Cattle: 10% (1 of 10) Sheep: 0 Goat: 0	Cattle: 50% (5 of 10) Sheep: 50% (5 of 10) Goat: 60% (3 of 5)
Parsabad	Cattle: 11.1% (1 of 9) Sheep: 0 Goat: 20% (1 of 5)	Cattle: 0 Sheep: 0 Goat: 0	Cattle: 33.3% (3 of 9) Sheep: 20% (2 of 10) Goat: 20% (1 of 5)	Cattle: 0 Sheep: 10% (1 of 10) Goat: 0	Cattle: 11.1% (1 of 9) Sheep: 0 Goat: 0	Cattle: 22.2% (2 of 9) Sheep: 0 Goat: 0	Cattle: 0 Sheep: 0 Goat: 20% (1 of 5)	Cattle: 0 Sheep: 0 Goat: 0	Cattle:0 Sheep: 10% (1 of 10) Goat: 0
Sareyn	Cattle: 0 Sheep: 0 Goat: 0	Cattle: 0 Sheep: 0 Goat: 0	Cattle: 12.5% (1 of 8) Sheep: 22.2% (2 of 9) Goat: 0	Cattle: 37.5% (3 of 8) Sheep: 11.1% (1 of 9) Goat: 33.3% (1 of 3)	Cattle: 12.5% (1 of 8) Sheep: 0 Goat: 0	Cattle: 0 Sheep: 0 Goat: 0	Cattle: 25% (2 of 8) Sheep: 11.1% (1 of 9) Goat: 33.3% (1 of 3)	Cattle: 0 Sheep: 0 Goat: 0	Cattle: 0 Sheep: 0 Goat: 0
*p*-value	0.813	0.010	0.031	0.461	0.719	0.727	0.897	0.162	0.162
Total	9.2% (26)	1% (3)	11.3% (32 of 281)	7.8% (22 of 281)	8.1% (23 of 281)	6.4% (18 of 281)	3.9% (11 of 281)	3.2% (9 of 281)	12.8% (36 of 281)

## Discussion

It is now widely recognized that human health is intrinsically linked to the health of animals, plants, and the environment. In this sense, intrinsically linked to food safety, it depends on knowing the pathogens that occur in the foods that are produced. Without understanding the prevalence of foodborne pathogens, it will be challenging to prioritize mitigation measures, resulting in suboptimal improvements in food safety [16]. Estimates suggest that up to one-third of the population in developed countries experiences a foodborne disease each year, with the prevalence likely being even higher in developing nations [17]. *C. burnetii*, an intracellular Gram-negative coccobacillus, is the causative agent of Q fever, which is a foodborne disease and public health problem in many countries including Iran [18, 19]. According to a systematic review and meta-analysis, molecular prevalence of *C. burnetii* in raw bulk milk of cattle, sheep, and goat in Iran was 15.1, 3.8, and 7.8%, respectively [18]. The frequency of *C. burnetii* in various bulk milk samples in Ardabil province was as follows: cattle 9.4%, sheep 7.9%, and goat 13.3%. These differences in *C. burnetii* frequency could be explained by herd size and density of animals, the presence of ticks, and bacterial shedding patterns from ruminants. The important shedding route for cattle is milk, while for small ruminants is parturition products [19].

On the other hand, the frequency of *C. burnetii* was notably higher in the Mughan region, characterized by its low altitude and warm climate—including Bilehsavar, Germi, Parsabad, Jafarabad, and Aslanduz—compared to the colder, high-altitude areas of the province. It is worth mentioning, the overall molecular frequency of *C. burnetii* in bulk milk samples in this study was 9.2%. Similar result in accordance with our study reported in Latvia (10.7%), but it was higher in South Korea (17.8%), Netherlands (18.8%), Portugal (20%), UK (25.9%), Belgium (30%), Italy (40.2%), Colombia (45.5%), Spain (51.7%), Italy (60.3%), USA (61.1%), and Hungary (66.7%) [19]. Uncertainty about the importance of milkborne infection could be a possible reason for higher prevalence in high-income countries, which resulted in a lack of control or different/weak control measures of *C. burnetii* in bulk milk samples [19].

*L. monocytogenes*, an intracellular Gram-positive pathogen, is the main causative agent of a sporadic foodborne disease called listeriosis. Poor milking hygiene, substandard storage conditions, and unsafe transport practices can contribute significantly to the contamination of milk with *L. monocytogenes*. A systematic review and meta-analysis revealed a low prevalence of *L. monocytogenes* in food samples, including milk, in Iran (4%) [20, 21]. In addition, the average prevalence of *L. monocytogenes* in milk in the USA was estimated at 4.3% [22]. The contamination of milk samples with *L. monocytogenes* in Ardabil was lower (1%). Also, this bacterium was exclusively found in milk samples from the province’s northernmost region (Aslanduz, with a warmer climate) and its southernmost area (Khalkhal, with a colder climate). Despite Ardabil’s cold climate favoring *L. monocytogenes*, the low frequency in Ardabil could reflect a genuine low environmental burden, possibly due to limited contamination sources, effective hygiene practices, or ecological suppression. However, methodological limitations, particularly the sensitivity of *hlyA*-based detection, cannot be ruled out. A combination of multiplex PCR, culture-based confirmation, and seasonal sampling would strengthen future surveillance.

Brucellosis is another foodborne disease can be transmitted by consuming unpasteurized/raw dairy products [23]. Despite ongoing efforts to eradicate brucellosis in Iran, the disease remains endemic and significantly affects ruminants, leading to substantial economic losses. Given the shared grazing areas and seasonal livestock migration, assessing *Brucella* transmission among herds and its potential spread to humans is essential, particularly in the western regions of Iran, where raw dairy plays a vital role in local diets [24]. The prevalence of *Brucella* spp. in milk samples in Iran was 9.7% in a meta-epidemiological study [25]. The frequency of this milkborne pathogen in Ardabil province was 11.3%. As shown in Table 3, the presence of this bacterium’s DNA in milk samples from every city in the province suggests that brucellosis is endemic to the region. *B. melitensis* is the most significant *Brucella* species for public health, responsible for the most human brucellosis cases globally and commonly found in Iran [26]. However, this study could not determine the specific *Brucella* species isolated from unpasteurized milk samples, which was a limitation.

Unpasteurized raw milk is also a source of *Campylobacter* infections [27], with prevalence rates in Iran of 1% in farm-sampled milk and 3.3% in market-sampled milk [28]. The average prevalence of *Campylobacter* spp. in raw milk in the USA was estimated at 6% [22], which is comparable to our results in this study (7.8%). The widespread detection of *C. jejuni* DNA across most cities in the province highlights key risk factors for milk contamination, including raw milk consumption, cross-contamination during handling, waterborne exposure, inadequate milking hygiene, and improper storage conditions.

Cattle are a major source of human foodborne tuberculosis, primarily linked to the *M. tuberculosis* complex including *M. tuberculosis*, *M. bovis*, *M. africanum*, *M. canettii*, *M. caprae*, *M. microti*, and *M. pinnipedii*. These resilient microorganisms, mainly *M. bovis* and occasionally *M. tuberculosis*, are capable of surviving in milk and can be transmitted to human through milk and dairy products [4]. Around 50 million cattle worldwide are believed to be affected by *M. bovis*, and before pasteurization became widely adopted, it was responsible for 20 to 40% of human tuberculosis cases. It is important to note that tuberculosis resulting from *M. bovis* infection through milk consumption is typically extrapulmonary—such as lymphatic or gastrointestinal tuberculosis—rather than pulmonary. Today, the global infection rate is estimated at approximately 1.4% [29]. The frequency of these foodborne pathogens in cattle bulk milk samples in Ardabil province was 11.5%, which is similar to those reported by Haghi et al. in Zanjan (Iran) (13.3%) [4], and Franco et al. in Brazil (8%) [30]. However, Zahrakar et al. identified *Mycobacterium* species DNA in 26% of raw cattle milk samples in Lorestan, Iran [29]. The contamination of sheep milk with *M. tuberculosis* complex (Table 3) is likely influenced by multiple factors. Close interactions between infected cattle and sheep, shared grazing environments, and potential cross-species transmission create opportunities for bacterial spread. Furthermore, insufficient biosecurity protocols and poor hygiene during the milking process can exacerbate contamination risks, highlighting the need for improved disease control measures. A study identified Bilehsavar as having the highest population of nomadic tuberculosis patients in Ardabil province (54%) [31], which aligns with our findings that show the highest frequency of *M. tuberculosis* complex isolated from milk samples in Bilehsavar (47.8%). This suggests that milk may play a role in tuberculosis transmission from animals to humans.

*Salmonella* spp. are also zoonotic pathogens transmitted through the consumption of contaminated food and water. *Salmonella*-related infections have implications for public health and economic stability in both developed and developing nations [32]. The gastrointestinal tract of cattle can be an important shedding source of *Salmonella* spp [33]. These bacteria can survive in the environment and contaminate various foods, including milk during milking [33, 34]. Our study revealed a 6.4% contamination rate of *S. enterica* in milk samples. The results reported by other studies in Iran and overseas were as follows: Mazandaran 2%, Shahrekord 4%, and Egypt 1.5% [35, 36]. These differences may result from inadequate hygiene during milking, contaminated water sources in livestock farms, failure to meet maintenance and transportation standards, and exposure to infected animals.

*S. aureus* stands out as a well-known pathogen in food microbiology and is recognized as one of the most common causative agents for food poisoning among various bacteria [37]. These enterotoxigenic strains can contaminate various food sources, including dairy products [37]. In the present study, 3.9% of raw milk samples were contaminated with *S. aureus*. Studies reporting the prevalence of *S. aureus* in raw milk in different cities of Iran were as follows: Alborz (15.3 and 11%), Shahrekord (38.8%), Mashhad (18.4%), Khuzestan (11%), Chaharmahal va Bakhtiyari (27.5%), Mazandaran (12.8%), Tabriz (9%), Kurdistan (40.8%), Fars (11%), and Urmia (50%) [35]. Raw milk contamination with *S. aureus* can result from infected dairy cows, particularly those suffering from mastitis, or from unhygienic milking practices that introduce bacteria from the environment. Poor handling, inadequate refrigeration, and cross-contamination further facilitate bacterial proliferation, posing health risks to consumers. In this study, cattle’s milk exhibited a higher *S. aureus* contamination rate (5.7%) compared to sheep (1.7%) and goat milk (3.3%).

The major types of diarrheagenic *E. coli* are enteropathogenic *E. coli* (EPEC), enteroaggregative *E. coli* (EAEC), enterotoxigenic *E. coli* (ETEC), enteroinvasive *E. coli* (EIEC), and Shiga toxin-producing *E. coli* (STEC), often referred to as enterohemorrhagic strains (EHEC). A notable genetic marker shared between EPEC and STEC is the *eaeA* gene, which encodes the protein responsible for the attaching and effacing mechanism in host cells [14, 38]. A meta-analysis estimated the prevalence of STEC serotypes in cattle in Iran to range between 3.1 and 6.3%. Despite data indicating that Iran is a high-risk region for STEC transmission, the organism is not recognized as a health hazard. As a result, the illness is not included in the contagious disease catalogue of the Iranian Ministry of Health and Medical Education, and no surveillance measures have been established in the country [39]. A research by Madani et al. conducted in Isfahan, Iran, reported that 37% of the isolates (20 out of 54) were classified as EPEC, while 7.4% (4 out of 54) were identified as STEC [38]. Other studies employed a combination of genetic markers—including *eaeA* and *bfpA* for EPEC, and *Stx1*, *Stx2*, *eaeA*, *hlyA*, and *espP* for STEC—to achieve precise serotype identification [14, 38]. However, our study relied solely on detection of the *eaeA* gene, identifying 3.2% of isolates as diarrheagenic *E. coli*. Since this marker alone cannot differentiate between EPEC and STEC, the limited genetic profiling represents a significant constraint in our findings.

*B. cereus* spores are naturally exist in soil, water, and the environment and known to be common contaminants in food, capable of surviving high temperatures during cooking and pasteurization. In Iran, raw milk is the second most common source of *B. cereus* contamination (48.8%). This study identified *B. cereus* as the most prevalent bacterium contaminating milk (12.8%) [35]. *B. cereus* has the potential to cause clinical mastitis in cows [40], and its presence in raw milk samples in our study is likely attributable to either an infection in the bovine host or environmental contamination of milk by bacterial spores.

This study did not evaluate the correlation between milk contamination and human notifiable diseases. Consequently, we propose a One Health surveillance framework that integrates veterinary, food safety, and public health sectors through coordinated sampling, shared databases, molecular tracing, and joint risk assessments. Cross-sector collaboration and timely data exchange are essential for early detection of milk-borne pathogens and associated human illnesses, while coherent policies ensure sustainable control of zoonotic diseases.

## Conclusions

This study highlights the significant frequency of major foodborne pathogens in unpasteurized bulk milk samples from Ardabil province, Iran, underscoring the critical need for enhanced food safety measures. The high contamination rates, particularly of *B. cereus*, *Brucella* spp., and *C. burnetii*, pose serious public health risks. Implementing stringent monitoring and control strategies in the dairy industry is essential to reduce the incidence of foodborne diseases and protect consumers. As a practical policy step, piloting on-farm interventions such as subsidized pasteurizers and hygiene training programs could significantly lower contamination risks at the source. Continuous surveillance and education on proper food handling practices are imperative to safeguard public health and prevent future outbreaks of foodborne illnesses in the region.

## Data Availability

The datasets generated and analyzed during the current study are available in the NCBIGenBank repository, under the accession numbers: PV344576 to PV344581.
